# Spirometer

**Published:** 1876-07

**Authors:** 


### *SPIROMETER.*

Doctor Hall always maintained that the lungs required vigorous and full exercise to promote health and ensure them against disease, and that this exercise should be one of the daily duties of life for persons with weak lungs. He believed that the "spirometer" was the only instrument adapted to lung exercise, and regretted that the cheapest "spirometer" in the market cost $10—a price beyond the means of many who require them. He used to try to devise some cheaper thing to accomplish the result, and in a recent number of the JOURNAL recommended the use of a rubber "life preserver" as the cheapest thing that would answer the purpose. But the "life preserver" costs $4, and is at best a very imperfect "spirometer." Only two days before the death of Dr. Hall he had given his hearty approval of a newly-invented "spirometer," which, he declared, fulfilled all the conditions of a complete instrument.

One of the most important of these conditions is its price—five dollars—which brings it within reach of nearly every one. This instrument is to be known as "Dr. Hall's Spirometer."

It is compact, and may be carried as conveniently as a book of medium size, or sent by mail, the postage being only a few cents. It is always ready for use, requiring no adjustment, and indicates on a scale the quantity of air expelled from the lungs.

The great advantage of such an instrument is that it keeps us informed as to the condition of the lungs and their daily progress toward healthy action. The capacity of weak lungs is increased by the careful use of the "spirometer," and the moral effect upon the patient has often a value beyond price. When he finds that he can expel at least 240 inches of air from the lungs into the "spirometer" at a single expiration, he feels that he has left the ranks of the great army of consumptives. We are giving our personal attention to the manufacture of a large number of these instruments, and shall soon be ready to fill orders already received and to supply others ordering them.

One of these "spirometers" will be sent free by mail on receipt of $5.

Full instructions sent with every instrument. Address: HALL'S JOURNAL OF HEALTH, 137 Eighth Street, New York.

